# SEPATH: benchmarking the search for pathogens in human tissue whole genome sequence data leads to template pipelines

**DOI:** 10.1186/s13059-019-1819-8

**Published:** 2019-10-22

**Authors:** Abraham Gihawi, Ghanasyam Rallapalli, Rachel Hurst, Colin S. Cooper, Richard M. Leggett, Daniel S. Brewer

**Affiliations:** 1 Norwich Medical School, University of East Anglia, Bob Champion Research and Education Building, Norwich, NR4 7UQ UK; 2 Functional Crosscutting Genomics England Clinical Interpretation Partnership (GeCIP) Domain Lead, 100,000 Genomes Project, Genomics England, London, UK; 3 Norwich Research Park, Earlham Institute, Norwich, NR4 7UZ UK

**Keywords:** Metagenomics, Pipeline, Taxonomy, Classification, SEPATH, Cancer, Pathogens, Bioinformatics, Bacteria, Viral

## Abstract

**Background:**

Human tissue is increasingly being whole genome sequenced as we transition into an era of genomic medicine. With this arises the potential to detect sequences originating from microorganisms, including pathogens amid the plethora of human sequencing reads. In cancer research, the tumorigenic ability of pathogens is being recognized, for example, *Helicobacter pylori* and human papillomavirus in the cases of gastric non-cardia and cervical carcinomas, respectively. As of yet, no benchmark has been carried out on the performance of computational approaches for bacterial and viral detection within host-dominated sequence data.

**Results:**

We present the results of benchmarking over 70 distinct combinations of tools and parameters on 100 simulated cancer datasets spiked with realistic proportions of bacteria. mOTUs2 and Kraken are the highest performing individual tools achieving median genus-level F1 scores of 0.90 and 0.91, respectively. mOTUs2 demonstrates a high performance in estimating bacterial proportions. Employing Kraken on unassembled sequencing reads produces a good but variable performance depending on post-classification filtering parameters. These approaches are investigated on a selection of cervical and gastric cancer whole genome sequences where *Alphapapillomavirus* and *Helicobacter* are detected in addition to a variety of other interesting genera.

**Conclusions:**

We provide the top-performing pipelines from this benchmark in a unifying tool called SEPATH, which is amenable to high throughput sequencing studies across a range of high-performance computing clusters. SEPATH provides a benchmarked and convenient approach to detect pathogens in tissue sequence data helping to determine the relationship between metagenomics and disease.

## Background

The estimated incidence of cancer attributed to infection surpasses that of any individual type of anatomically partitioned cancer [1]. Human papillomavirus (HPV) causes cervical carcinoma, and *Helicobacter pylori* facilitates gastric non-cardia carcinoma induction [2, 3]. The role of HPV in tumorigenesis is understood and has clinical implications: HPV screening programs have been adopted and several vaccines exist, targeting a wide range of HPV subtypes [4]. The amount of whole genome sequencing data generated from tumor tissue is rapidly increasing with recent large-scale projects including The Cancer Genome Atlas (TCGA) Program [5], International Cancer Genome Consortium (ICGC) [6] (including the Pan-Cancer Analysis of Whole Genomes, PCAWG [7]), Genomic England’s 100,000 Genomes Project [8], and at least nine other large-scale national sequencing initiatives emerging [9]. When such samples are whole genome sequenced, DNA from any pathogens present will also be sequenced, making it possible to detect and quantify pathogens, as recently shown in cancer by Feng et al. [10] and Zapatka et al. [11]. Protocols for these projects do not typically encompass negative control samples and do not use extraction methods optimized for microbiome analysis, yet careful consideration of contamination and correlation of output results with clinical data could generate hypotheses without any additional cost for isolated metagenomics projects. The scope of potential benefits from analyzing cancer metagenomics is broad and could benefit multiple prominent research topics including cancer development, treatment resistance, and biomarkers of progression. It is therefore important to consider the performance of pathogen sequence classification methods in the context of host-dominated tissue sequence data.

Traditionally, the identification of microbiological entities has centered around culture-based methodologies. More recently, there has been an increase in taxonomic profiling by using amplicon analysis of the 16S ribosomal RNA gene [12]. Whole genome sequencing however presents an improved approach that can interrogate all regions of every constituent genome whether prokaryotic or not and provides a wider range of possible downstream analyses. The increasingly widespread use of whole genome sequencing technologies has resulted in an explosion of computational methods attempting to obtain accurate taxonomic classifications for metagenomic sequence data [13]. Typically, these tools rely on references of assembled or partially assembled genomes to match and classify each sequencing read or assembled contig. One issue with this approach is that there exists an uneven dispersion of interest in the tree of life, rendering some clades underrepresented or entirely absent. Furthermore, sequence similarity between organisms and contamination in reference genomes inhibit the perfect classification of every input sequence [14–16]. A recent study has shown that the increasing size of databases such as NCBI RefSeq has also resulted in more misclassified reads at species level with reliable classifications being pushed higher up the taxonomic tree [17]. Because of this species-level instability, we initially select to carry out metagenomic investigations at a genus level, prior to investigating lower taxonomic levels, particularly for experiments with low numbers of non-host sequences.

Computational tools for metagenomic classification can be generalized into either taxonomic binners or taxonomic profilers [13]. Taxonomic binners such as Kraken [18, 19], CLARK [20], and StrainSeeker [21] attempt to make a classification on every input sequence whereas taxonomic profilers such as MetaPhlAn2 [22, 23] and mOTUs2 [24, 25] typically use a curated database of marker genes to obtain a comparable profile for each sample. This generally means that taxonomic profilers are less computationally intensive in comparison with binners but may be less effective with low amounts of sequences. Although there is a large number of tools available purely for sequence classification, at the time of writing, there is a limited selection of computational pipelines available that process data optimally with high-throughput and produce classifications from raw reads with all appropriate steps including quality control. Examples of these include PathSeq [26–28] which utilizes a BLAST-based [29] approach and IMP [30] which utilizes MaxBin [31] for classification.

Community-driven challenges such as Critical Assessment of Metagenome Interpretation (CAMI) provide one solution to independently benchmark the ever-growing selection of tools used for metagenomic classification [13]. CAMI provides a useful starting point for understanding classification tools on samples with differing complexity, but it is unlikely to provide an accurate comparison for more niche areas of taxonomic classification such as ancient microbiome research [32] or for intra-tumor metagenomic classification dominated by host sequences.

Classifying organisms within host tissue sequence data provides an additional set of challenges. In addition to the limitations in the tool performance, there is also a low abundance of pathogenic sequences compared to the overwhelming proportion of host sequence data as well as high inter-sample variability. Cancer sequences are also known to be genetically heterogeneous and unstable in nature providing a further cause for caution when classifying non-host sequences and rendering the accurate removal of host reads difficult [33–35].

Here, we present and discuss the development of SEPATH, template computational pipelines designed specifically for obtaining classifications from within human tissue sequence data and optimized for large WGS studies. This paper provides rationale for the constituent tools of SEPATH by analyzing the performance of tools for quality trimming, human sequence depletion, metagenomic assembly, and classification. We present the results of over 70 distinct combinations of parameters and post-classification filtering strategies tested on 100 simulated cancer metagenomic datasets. We further assess the utility of these pipelines by running them on a selection of whole genome cancer sequence data. We analyze a selection of samples from cervical cancer, where it is expected that *Alphapapillomavirus* will be frequently identified and gastric cancer where it is expected that *Helicobacter* will be identified. A selection of 10 pediatric medulloblastoma samples is also analyzed for which it is expected that not many if any taxa at all will be identified due to the historically noted sterility of the brain, although this is currently a subject of debate within the scientific community [36].

## Results

The process of obtaining pathogenic classifications from host tissue reads can be broken down into a few key computational steps: sequence quality control, host sequence depletion, and taxonomic classification. For these computational steps, a series of tools and parameters were benchmarked on simulated metagenomes (see the “Methods” section). These genomes emulate empirical observations from other cancer tissue sequence data [11], with the percentage of human reads ranging from 87 to > 99.99%. Genomes from 77 species were selected as constituents for the metagenomes [37]. These species were identified from Kraal et al. [38] with additional bacterial species associated with cancer, e.g., *Helicobacter pylori* [2] (see Additional file 1 for a full description of each simulation).

### Human sequence depletion

A large proportion of sequence reads from tumor whole genome sequencing datasets are human in origin. It is essential to remove as many host reads as possible—firstly, to limit the opportunity for misclassification and, secondly, to significantly reduce the size of data thereby reducing the computational resource requirement.

Three methods of host depletion were investigated on 11 simulated datasets (2 × 150 bp Illumina reads). Two of these methods were *k*-mer-based methods: Kontaminant [39, 40] and BBDuk [41]. The third method involved extracting unmapped reads following BWA-MEM [42] alignment, an approach that is facilitated by the likelihood that data will be available as host-aligned BAM files in large-scale genomic studies. BWA-MEM is used as a baseline, and parameters were set to be as preservative as possible of any potential non-human reads.

**Fig. 1:**
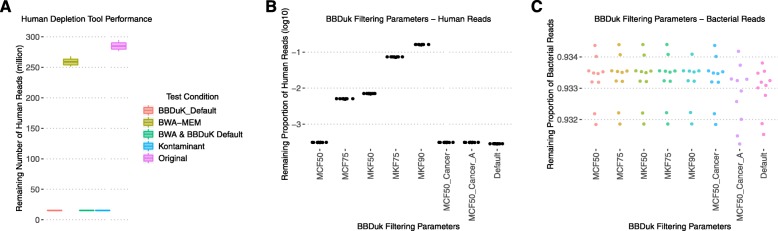
Human read depletion performance. **a** Human read removal using BBDuK, BWA-MEM, and Kontaminant. The remaining numbers of human reads were near identical for BBDuK and Kontaminant (median values of 15,399,252 and 15,399,928 for BBDuK and Kontaminant, respectively.) All conditions retained bacterial reads with near-identical performance (Additional file 2: Figure S1). BBDuK was selected for parameter optimization (**b**, **c**). This analysis was performed on raw untrimmed reads of *n* = 11 simulated datasets. **b**, **c** BBDuk parameter optimization in terms of the remaining human reads (**b**) and remaining bacterial reads (**c**). Default BBDuK settings were used along with alterations of MKF and MCF parameters. The default parameters of BBDuK remove a sequencing read in the event of a single *k*-mer match, whereas MCF50 requires 50% of the bases in a read to be covered by reference *k*-mers for removal and MKF50 requires 50% of *k*-mers in a read to match the reference for removal. MCF50-Cancer indicates that BBDuK was ran with a database consisting of GRCh38 human reference genome and a collection of known mutations in human cancer from the COSMIC database. MCF50_Cancer_A denotes a database consisting of human reference genome 38, COSMIC cancer genes, and additional sequences from a recent African “pan-genome” study [44] (**b**). Default and both MCF50 parameters (with and without cancer sequences) showed the highest removal of human reads

All methods retained the majority of bacterial reads (median of > 99.9% bacterial reads retained for all conditions; Additional file 2: Figure S1), but the number of human reads remaining in each dataset varied (Fig. 1). Using default parameters, BBDuK and Kontaminant retained a median of 15.4 million reads, compared to 259 million from BWA-MEM with intentionally lenient filtering parameters. We investigated BBDuK further, establishing default BBDuK performance following BWA-MEM depletion which demonstrated no tangible difference in human read removal (Fig. 1a). BBDuK parameters were also adjusted from the default setting of a single *k*-mer match to the reference database (Fig. 1b, c). It was found that removing a read when 50% or more of the bases have *k*-mer matches to the human reference (MCF50) provided an approach that removed near-identical proportions of human and bacterial sequences to the default parameters.

In an attempt to capture *k*-mers specific of cancer sequences, a BBDuK database was generated containing human reference genome 38 concatenated with coding sequences of all cancer genes in the COSMIC database [43]. With the additional cancer sequences, a near-identical performance was obtained when compared with just the human reference database (Fig. 1b, c). Therefore, including extra cancer sequences did not alter the retention of pathogen-derived reads, providing an opportunity for increased human sequence removal on real data without sacrificing bacterial sensitivity. To investigate using a BBDuK database capturing a higher degree of human sequence variation, we also investigated the inclusion of additional human sequences from a recent analysis into the African “pan-genome” [44]. Including these extra sequences removed slightly more bacterial reads but this had a very minor effect (Fig. 1c).

### Taxonomic classification: bacterial datasets

**Fig. 2:**
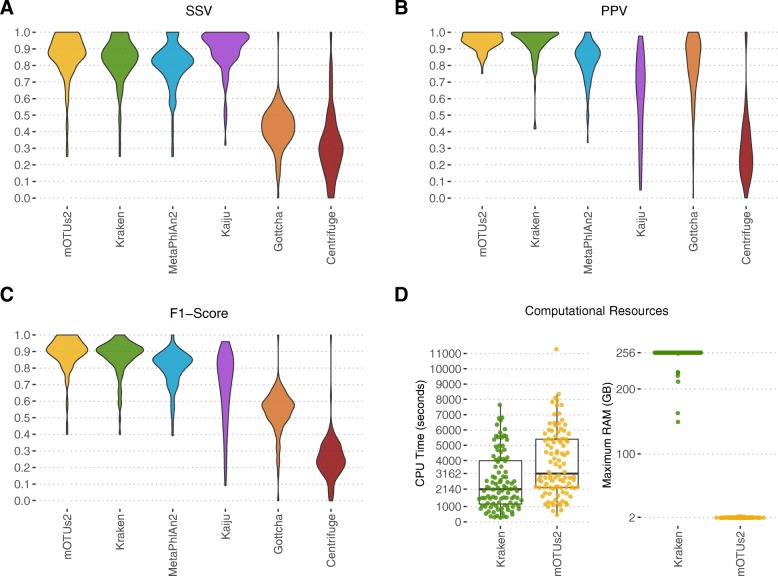
Performance estimates for taxonomic classification tools. Methods were applied to quality filtered and human-depleted sequencing reads on 100 metagenome simulations. Performance is summarized at the genus level in terms of sensitivity (**a**), positive predictive value (**b**), and F1 score (**c**). Computational resources in terms of CPU time and RAM is also shown for the top two performing tools: Kraken and mOTUs2 (**d**). Kraken utilized 20 threads for most datasets whereas mOTUs2 utilized 17. mOTUs2 output was unfiltered, whereas Kraken had a confidence threshold of 0.2 and a subsequent read threshold of 500 applied to determine positive classifications. Parameters for each tool in this graphic were selected from the top-performing parameters observed for multiple tests with varying parameters

We compared the performance of 6 different taxonomic classification tools by applying them after filtering and host depletion on 100 simulated datasets. Performance was measured in terms of presence/absence metrics at the genus level: positive predictive value (PPV/precision), sensitivity (SSV/recall), and F1 score (the harmonic mean of precision and recall). Sequences were classified using 3 taxonomic profilers (mOTUs [25], MetaPhlAn2 [22, 23], and Gottcha [45]) and 3 taxonomic binners (Kraken [18], Centrifuge [46], and Kaiju [47]) (Fig. 2a–c). In our analysis, Kraken and mOTUs2 delivered the best median genus F1 of 0.90 (IQR = 0.083) and 0.91 (IQR = 0.10), respectively, with median genus PPV scores of 0.97 (IQR = 0.084) and 0.95 (IQR = 0.080), and median genus sensitivity scores of 0.86 (IQR = 0.123) and 0.88 (IQR = 0.126) for Kraken and mOTUs2, respectively.

Kraken utilizes over 125 times the RAM requirement of mOTUs2 (Fig. 2d; median 256 GB vs 2 GB RAM for Kraken and mOTUs2, respectively; *p*=2.2×10^−16^ Mann-Whitney *U* test); Kraken was ran with the database loaded into RAM to improve runtime. Historically, alignment-based taxonomic classification tools have been slow, but by using the reduced 40 marker gene database, mOTUs2 has much lower run times. CPU time was on average marginally higher for mOTUs2 compared to Kraken (Fig. 2d), but we noticed the elapsed time was actually lower (data not shown).

### Bacterial proportion estimation

**Fig. 3:**
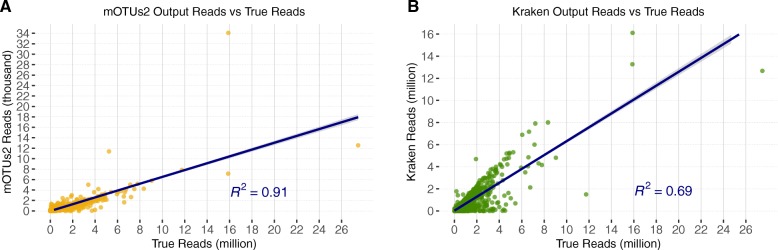
Quantitative ability for mOTUs2 and Kraken. mOTUs2 output reads vs true reads (**a**) and Kraken output reads vs true reads (**b**). For all true-positive genera classifications (Spearman’s rank correlation coefficients *R*^2^=0.91 and *R*^2^=0.69, for *n* = 2084 and *n* = 2021 true-positive classifications for mOTUs2 and Kraken, respectively). All 100 simulated datasets were first quality trimmed using Trimmomatic and depleted for human reads using the best parameters as previously mentioned. mOTUs2 classifications were left unfiltered whereas Kraken had a confidence threshold of 0.2 and a minimum read threshold of 500 applied

Analyzing population proportions can provide a deeper understanding of microorganism community structure. Therefore, it is important to assess the performance of tools in predicting proportions. For each true-positive result from the top-performing pipelines using Kraken and mOTUs2, the output number of reads was compared against the true number of reads in the simulations (Fig. 3). The mOTUs2 pipeline obtained accurate rankings of read estimates (*R*^2^ = 0.91; Spearman’s rank-order correlation) whereas our Kraken pipeline predicted the number of reads with Spearman’s rank-order correlation value of *R*^2^ = 0.69.

### Bacterial classification following metagenomic assembly

The data above demonstrates that mOTUs2 and Kraken have comparable performances. However, Kraken, in contrast to mOTUs2, can classify non-bacterial sequences. When ran on raw reads, Kraken typically requires post-classification filtering strategies in order to obtain high performance [25] (Additional file 3: Figure S2). Post-classification filtering involves applying criteria to remove low-quality classifications from taxonomic results. Applying a metagenomic assembly algorithm to quality-trimmed non-host reads might provide a rapid filtering approach that reduces the need for read-based thresholds.

**Fig. 4:**
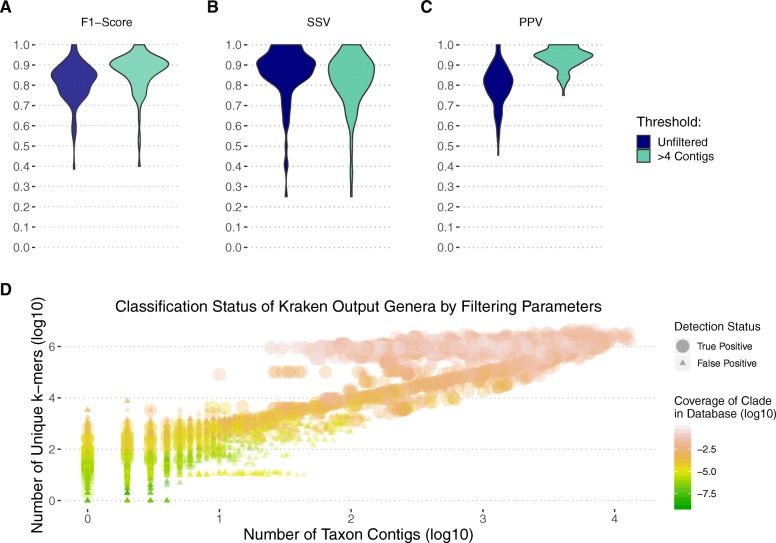
Genus-level performance of Kraken on contigs following metagenomic assembly with MetaSPAdes. Performance is summarized by genus-level F1 score (**a**), sensitivity (**b**), and PPV (**c**). A single dataset failed metagenomic assembly, and so, data shown is for 99 of 100 simulated datasets. Performance is shown on raw Kraken classifications with no threshold applied (unfiltered) in dark blue. The light blue is the performance when a minimum of 5 contigs assigning to a genera was used. Median values for unfiltered performance were 0.83, 0.88, and 0.81, and for filtered performance were 0.89, 0.85, and 0.94 for F1 score, sensitivity, and PPV, respectively. **d** KrakenUniq filtering parameters in relation to detection status. The *y*-axis indicates the number of unique *k*-mers assigned to a particular taxon, the *x*-axis represents the number of contigs assigned to a particular taxon (log10), and the color gradient shows the coverage of the clade in the database (log10). True-positive results are larger circles, whereas false-positive results are smaller triangles. The scatter plot shows 10,450 contigs classified at genus level as data points; the ggplot package alpha level was set to 0.3 due to a large number of overlapping points. *k*= 31

MetaSPAdes [48] was employed on high-quality non-human reads from 100 simulated datasets. An F1 score of 0.83 was obtained without any read threshold, which was an improvement over Kraken on raw reads without any filtering strategies (F1 = 0.54) but lower than Kraken with filtering (F1 = 0.9). The F1 score was increased to 0.89 when a requirement for a minimum of 5 classified contigs for classification was applied (Fig. 4a). Filtering out contigs with lower coverage made little difference on the performance with the parameters tested (Additional file 4: Figure S3, Additional file 5: Figure S4).

Filtering these datasets by number of contigs is non-ideal, as it would remove classifications from taxa that assembled well into a small number of contigs. An evolution of Kraken, KrakenUniq [19], was run on these contigs to further illuminate the relationship between taxa detection and more advanced metrics than Kraken 1, including the coverage of the clade in the reference database and the number of unique *k*-mers (Fig. 4d, Additional file 6: Figure S5). This analysis reveals that on our challenging datasets, no set of filtering parameters could obtain perfect performance. Upon investigation of a single dataset, it was observed that 13 out of 17,693 contigs assigning to different genera were responsible for false-positive classifications resulting in a drop of PPV to 0.83 (Additional file 7: Figure S6). These contigs were extracted and used as input for NCBI’s MegaBLAST with standard parameters. Of the 13 false-positive contigs, 3 were correctly reclassified, 3 were incorrectly classified, and the remaining 7 obtained no significant hits. This highlights that these contigs may suffer from misassembly or non-uniqueness that is not improved by use of a tool with a different approach.

### Taxonomic classification: viral datasets

**Fig. 5:**
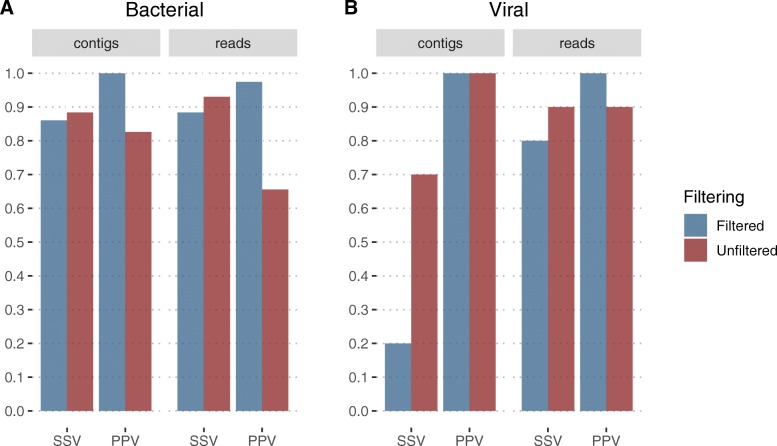
Kraken performance on a single dataset containing both bacterial (**a**) and viral (**b**) reads. Performance from metagenomic assembly approach is shown on both unfiltered contigs and results filtered by a minimum of 5 contigs required for classification. Kraken performance on raw reads is shown both unfiltered and filtered by a minimum of 100 reads for classification. Bacterial performance is classified at genus level whereas viral performance is regarding species level due to peculiarities in taxonomy

We established the performance of viral classification in the presence of bacterial noise by spiking a selection of our host-bacterial datasets with 10,000 viral reads for each 10 species. As mOTUs2 does not make viral classifications, Kraken was run on either quality-trimmed reads or contigs following metaSPAdes [48] assembly (see the “Methods” section). Kraken correctly identified 8/10 virus species from reads as input with post-classification filtering. When using contigs and no filtering strategies, 7/10 species were detected with no viral false-positive results (Fig. 5b). Filtering by minimum number of contigs removed the majority of viral classifications. The effect of filtering on viral species classification was not reflected in the classification of bacterial genera (Fig. 5a).

### Bacterial consensus classification

**Fig. 6:**
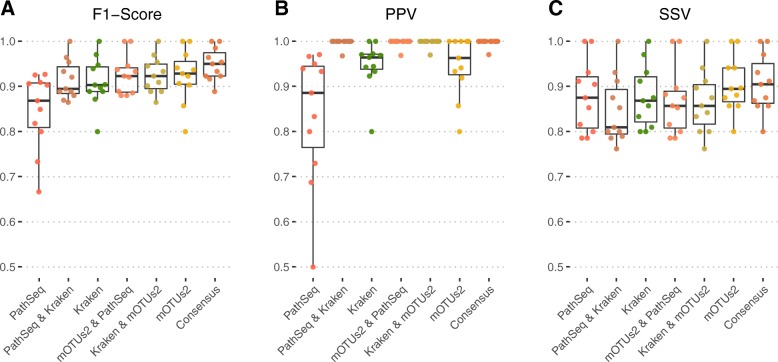
mOTUs2, Kraken, and Pathseq form a consenus with near-perfect genus-level classification performance. Box plots with individual data points for *n* = 11 simulated bacterial metagenomes showing genus-level F1 score (**a**), PPV (**b**), and SSV (**c**) for single tools, an intersection of classification between two tools, and a consensus of all three tools. PPV obtained perfect values in the result of an intersection between two tools or a consensus. Sensitivity generally decreases in the event of combining two tools with an intersection but increases to a median score of 0.905 in the result of an intersection. This raise in sensitivity resulted in a genus-level F1 score in the consensus approach of 0.95. mOTUs2 output files were unfiltered, whereas Kraken had a filter of >4 contigs and PathSeq >1 reads

Using distinct methods of classification and combining the results have been shown to improve metagenomic classification performance [49]. The Kraken/mOTUs2 pipelines outlined here were compared with the BLAST-based [29] PathSeq [27, 28] on a reduced selection of 11 simulated bacterial datasets (Fig. 6). A smaller selection of datasets was used due to local resource limitations in terms of storage and computational time of aligning our simulations to the human genome to produce the required input for PathSeq. It was found that using an intersection of classifications between any two tools obtained a perfect median PPV score but caused a small drop in sensitivity and resulted in similar F1 scores compared with using single tools. Sensitivity increased to 0.905 when using a consensus approach between all three tools (whereby classifications made by at least 2/3 tools is taken as true). This rise in sensitivity for the consensus approach resulted in a median genus-level F1 score of 0.95, which was a better score than any other single tool or intersection of two tools.

### Real cancer whole genome sequence data

**Fig. 7:**
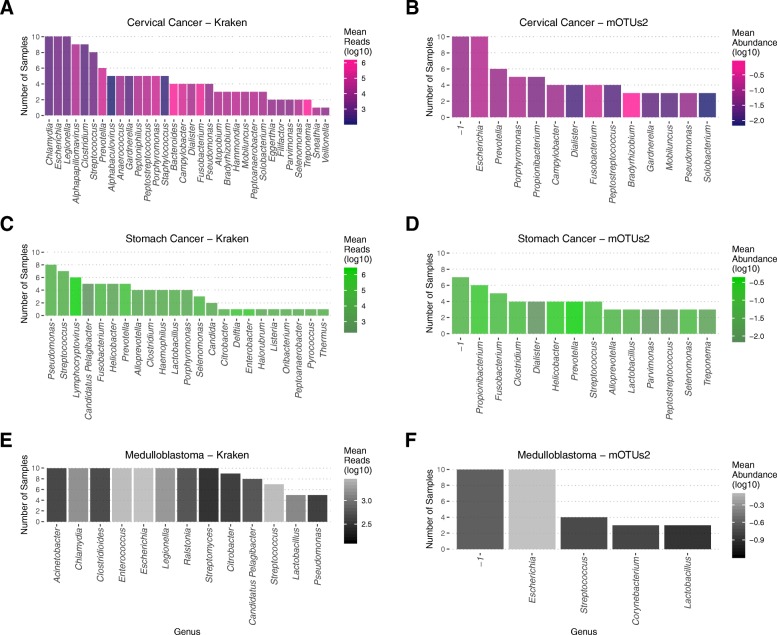
The application of SEPATH pipelines on a range of cancer types. Output genera from Kraken (left) and mOTUs2 (right) human-depleted, quality-trimmed reads from whole genome sequencing files. *n* = 10 for each of cervical cancer (**a**, **b**), stomach cancer (**c**, **d**), and medulloblastoma (**e**, **f**). For display purposes, mOTUs2 results were filtered to show taxa that occurred in at least 3 samples. Kraken results were filtered for taxa that were in a minimum of 5 samples, or had a mean read count of over 5,000

SEPATH pipelines using Kraken and mOTUs2 were ran on quality-trimmed, human-depleted sequencing files (Fig. 7). Kraken identified *Alphapapillomavirus* to be present in 9/10 cervical squamous cell carcinoma samples, with a high average number of sequencing reads compared to other taxa (Fig. 7a). Interestingly, *Treponema* was identified as present in two samples by both techniques (taxa detected in ≥3 samples displayed in Fig. 7b), and both tools report high quantitative measures. This may well represent an interesting diagnostic finding, although follow-up would be required to ascertain the clinical utility. In stomach cancer, both mOTUs2 and Kraken identified *Helicobacter* in 4 and 5 samples, respectively, as anticipated; Kraken reported *Lymphocryptovirus*in 6/10 samples with a high number of reads in addition to a variety of other genera (Fig. 7c). Despite human read depletion, care should be taken to ensure the true-positive nature of *Lymphocryptovirus* as has been reported [50, 51]. It is noteworthy that the classification is not prominent in either cervical cancer or medulloblastoma and has previously been associated with gastric oncogenesis [3, 52].

In both cervical and gastric cancers, expansion of these pipelines to larger datasets would help to characterize the role of many other reported genera. Medulloblastoma samples are expected to be mostly sterile, and this is well reflected with only very low number of genera at low read counts (number of genera: total reads in all samples 75: 11,213,997; 102: 16,269,893; 27: 138,712 for cervical, gastric, and medulloblastoma, respectively.). Kraken appears to be more sensitive, making a greater number of classifications overall and classifying the same taxa as present in a higher number of samples than mOTUs2.

### SEPATH template pipelines

**Fig. 8:**
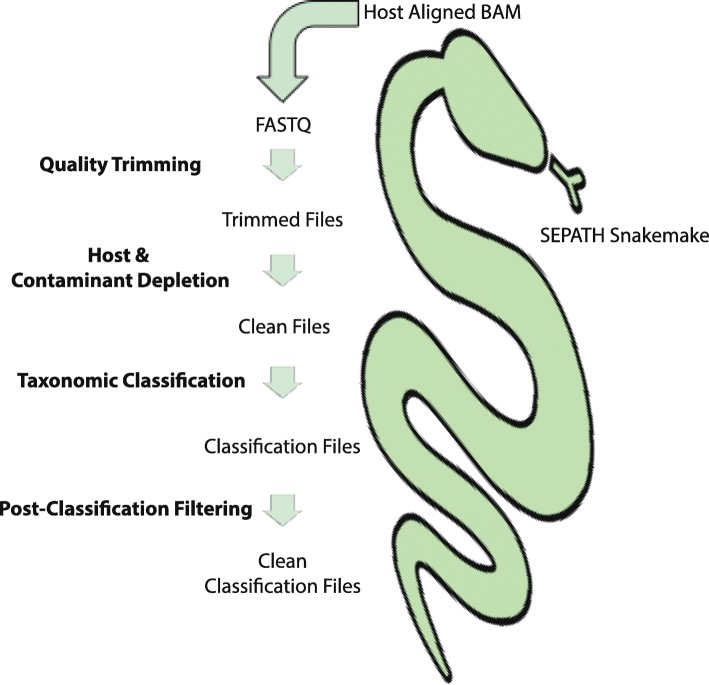
SEAPTH template computational pipeline. The top-performing pipelines from this benchmark are provided as a template for users to adjust according to their own job scheduling systems and resource availability. SEPATH provides two main pathways: a bacterial pipeline using mOTUs2 classifications on raw sequencing reads and a bacterial and viral pipeline employing Kraken on metagenomic contigs assembled using non-human reads with MetaSPAdes

The top-performing algorithms and parameters for each of the stages have been combined in a unifying template pipeline implemented in snakemake [53]: SEPATH (Fig. 8, https://github.com/UEA-Cancer-Genetics-Lab/sepath_tool_UEA). SEPATH provides three blocks of functionality: (1) conversion of host-aligned BAM files to FASTQ files that is intentionally preservative of pathogenic reads, (2) mOTUs2 bacterial classification ran on trimmed and filtered sequencing reads, and (3) Kraken ran on quality-trimmed reads or metagenomic-assembled contigs. All blocks can be run together or separately and uses either BAM of FASTQ input files. All software dependencies for SEPATH can easily be installed via conda.

## Discussion

We have demonstrated pipelines for detecting bacterial genera and viral species in simulated and real whole genome sequence data from cancer samples. These pipelines perform well in terms of sensitivity and PPV and utilize computational resources effectively. The two top-performing classification tools, Kraken and mOTUs2, have very different underlying mechanics despite achieving similar performance. Kraken builds a database by minimizing and compressing every unique *k*-mer for each reference genome. Kraken begins the analysis by breaking down each input read into its constituent *k*-mers and matching each of these to the user-generated reference database. The sequence is classified probabilistically by the leaf in the highest weighted root to leaf path in a taxonomic tree [18]. In comparison with Kraken, mOTUs2 uses a highly targeted approach by analyzing 40 universal phylogenetic bacterial marker genes for classification. Overall, mOTUs2 uses 7726 marker gene-based operational taxonomic units (mOTUs). Classifications are obtained by an alignment to this database using BWA-MEM with default parameters [25, 42].

mOTUs2 has been developed with quantitative abundance in mind. It intuitively estimates the proportion of sequences estimated to originate from unknown taxa (denoted by *“ − 1”* in mOTUs2 reports) and adjusts abundance values from detected clades accordingly to account for this. Kraken read distribution can be improved by using a Bayesian framework to redistribute the assigned reads using Bracken [54]. A comparison of relative abundance between mOTUs2 and Bracken was carried out during the production of mOTUs2 as reported in Milanese et al. [25], which demonstrated that mOTUs2 appeared to provide more accurate predictions. We therefore recommend our Kraken pipelines for accurate representations of presence/absence and suggest that using abundance weighted *β*-diversity metrics from these pipelines should be interpreted with caution. A further caveat of the assembly Kraken pipeline is that it requires successful metagenomic assembly. While MetaSPAdes worked well on our simulations, idiosyncrasies of differing technologies and datasets may hinder a successful assembly. In this event, we would recommend running Kraken classification on quality-trimmed and human-depleted sequencing reads without assembly.

The data in this paper supports the use of mOTUs2 for quantitative bacterial measurements, which together with the high classification performance on simulated data suggests that both binary and non-binary *β*-diversity measures would be representative of the true values of the dataset, suggesting a conferred accuracy in bacterial community profiling. Furthermore, mOTUs2 differs from the current methods that rely purely on bacterial reference sequences by incorporating data from metagenome-assembled genomes, suggesting that mOTUs2 captures a differing scope of classifications to our Kraken database, which was developed using reference genomes. Although both tools are state-of-the-art at the time of writing, they are likely to contain biases in terms of what they are able to classify, which pertains to previous sequencing efforts of the sampling site. The human gut microbiome for example is currently believed to be better characterized than other body sites [25].

For bacterial classification, we noted a higher performance at taxonomic levels above genus level, but performance appears to drop at species level (Additional file 3: Figure S2). We urge caution when working at the species level on this type of data due to this combined with the instability of species-level classification. At lower taxonomic levels, the retention of BAM files from mOTUs2 could theoretically allow for subsequent investigations at more specific taxonomic nodes (such as strain level) by investigating single-nucleotide variation. Kraken also automatically produces subgenus-level classifications where the input data and reference database permits. Validating performance at these taxonomic levels would require extensive performance benchmarking which has not been conducted here. Benchmarking tools and databases as they emerge are important tasks as they greatly influence performance. It is hoped that utilities presented here will assist future benchmarking efforts.

The use of SEPATH pipelines on real cancer sequence data suggests overall agreement between Kraken and mOTUs2 but reveals important considerations for subsequent analysis. Kraken appears to be more sensitive than mOTUs in this real data, possibly due to the differing parameters used due to the shorter read lengths seen (2 × 100 bp in real sample data compared to 2 × 150 bp in simulated data). Using sequencing protocols optimized for microbial detection compared to human sequencing projects is likely to result in a higher and more even microbial genome coverage and subsequently more classifications with mOTUs2 which has been demonstrated recently in the analysis of fecal metagenomes of colorectal cancer patients [55]. In this study, mOTUs2 provided interesting “unknown” classifications which would not be captured by standard Kraken databases. We therefore recommend Kraken as the primary tool of investigation on tissue, but mOTUs2 has a great potential in the confirmatory setting and for investigating unknown taxa. A consensus approach of different tools on much larger real datasets would likely help in distinguishing between the peculiarities (particularly false positives) of individual tools and true-positive results which would benefit the accurate characterization of human tissue metagenomes.

## Conclusions

A benchmark into metagenomic classification tools has revealed high-performing approaches to process host-dominated sequence data with low pathogenic abundance on a large selection of challenging simulated datasets. We provide these pipelines for the experienced user to adjust according to their own resource availability and provide our simulated metagenomes for others to use freely for independent investigations. mOTUs2 provides fast and accurate bacterial classification with good quantitative predictions. MetaSPAdes and Kraken provide bacterial and viral classification with assembled contigs as a useful downstream output. We have shown that SEPATH forms a consensus alongside PathSeq to achieve near-perfect genus-level bacterial classification performance. Using SEPATH pipelines will contribute towards a deeper understanding of the cancer metagenome and generate further hypotheses regarding the complicated interplay between pathogens and cancer.

## Methods

### Metagenome simulations

Metagenomes were simulated using a customized version of Better Emulation for Artificial Reads (BEAR) [56] and using in-house scripts to generate proportions for each reference genome (Additional file 8: Figure S7, https://github.com/UEA-Cancer-Genetics-Lab/BEAR). These proportions were based on previously analyzed cancer data [11]. Firstly, the number of total bacterial reads (in both pairs) was generated by a random selection of positive values from a normal distribution function with a mean of 28,400,000 and a standard deviation of 20,876,020. The number of human reads in the sample was set to the difference between this number and 600 million (the total number of reads in both pairs). The number of bacterial species was randomly sampled from the reference species available, and the number of bacterial reads available was picked from a gamma distribution of semi-random shape. The number of reads for each bacterial species was distributed among contigs proportionately depending on the contig length. This produced a file with contigs and proportions of final reads which was provided to BEAR to generate paired-end FASTA files for each of the 100 metagenomes with approximately 300 million reads per paired-end file (complete metagenome compositions can be found in Additional file 1, viral components in Additional file 9). An error model was generated following the BEAR recommendations from a sample provided by Illumina containing paired-end reads that were 150 bp in read length (https://basespace.illumina.com/run/35594569/HiSeqX_Nextera_DNA_Flex_Paternal_Trio). This sample was selected to best resemble data originating from within Genomic England’s 100,000 Genomes Project. These simulated metagenomes can be downloaded from the European Nucleotide Archive (https://www.ebi.ac.uk/ena/data/view/PRJEB31019).

### Tool performance benchmarking

Samples were trimmed for quality, read length, and adapter content with Trimmomatic [57] prior to running any classification (default parameters were minimum read length = 35 and minimum phred quality of 15 over a sliding window of 4). SEPATH has trimming parameters set as default that prevent any excessive removal of data (including any reads that may be pathogenic), but these should be adjusted according to the nature of the data being analyzed.

Performance estimates were obtained by converting all output files into a common file format which were compared against the true composition by string matches and NCBI taxonomic ID. The total number of true-positive results, false-positive results, and false-negative results was used to calculate F1 score; sensitivity and PPV were calculated as follows:

1 $$  {\mathrm{SSV (recall) = \frac{TP}{TP + FN}}}  $$

2 $$  {\mathrm{PPV (precision) = \frac{TP}{TP + FP}}}  $$

3 $$  {\mathrm{F1-score = \frac{2}{SSV^{-1} + PPV^{-1}}}}  $$

### Real cancer whole genome sequence analysis

Sequencing data from cancer tissue was obtained from The Cancer Genome Atlas (TCGA-CESC and TCGA-STAD) [5], International Cancer Genome Consortium (ICGC) PedBrain Tumor Project [58], and ICGC Chinese Gastric Cancer project [59]. These sequencing reads were pre-processed through a common pipeline to obtain reads unaligned to the human genome [60] and were additionally quality trimmed and depleted for human reads using SEPATH standard parameters but with a database consisting of human reference genome 38, African “pan-genome” project sequences and COSMIC cancer genes as previously mentioned. Kraken was ran on quality-trimmed reads, and a confidence threshold of 0.2 was applied to the reports. mOTUs2 was ran for the genus-level analysis on the same reads using 2 marker gene minimum and a non-standard minimum alignment length of 50 to account for shorter read length. Kraken files had a minimum read threshold applied of 100 reads for each classification, and mOTUs2 results were left unfiltered.

### Computational tools and settings

All analysis for figures was carried out in R version 3.5.1 (2018-07-02). All scripts and raw data used to make the figures can be found in the supplementary information and on https://github.com/UEA-Cancer-Genetics-Lab/sepath_paper. In addition to the “other requirements” mentioned below, this paper used the following software as part of the analysis: picard 2.10.9, samtools v1.5, BEAR (https://github.com/UEA-Cancer-Genetics-Lab/BEAR commit: a58df4a01500a54a1e89f42a6c7314779273f9b2), BLAST v2.6.0+, Diamond v0.9.22, MUMmer v3.2.3, Jellyfish v1.1.11, Kaiju v1.6.3, Kontaminant (pre-release, GitHub commit: d43e5e7), KrakenUniq (github commit: 7f9de49a15aac741629982b35955b12503bee27f), MEGAHIT (github commit: ef1bae692ee435b5bcc78407be25f4a051302f74), MetaPhlAn2 v2.6.0, Gottcha v1.0c, Centrifuge v1.0.4, FASTA Splitter v0.2.6, Perl v5.24.1 bzip2 v1.0.5, gzip v1.3.12, and Singularity v3.2.1.

Python v3.5.5 was used with the exception of BEAR, which used Python 2.7.12. Python modules used the following: SeqIO of BioPython v1.68, os, sys, gzip, time, subprocess, and glob. The following are the R packages used and their versions: Cowplot v0.9.3, dplyr v 0.7.6, ggExtra v0.8, ggplot2 v3.0.0, ggpubr v0.1.8, ggrepel v0.8.0, purr v0.2.5, ggbeeswarm v0.6.0, see v0.2.0.9, RColorBrewer v1.1-2, readr v1.1.1, reshape2 v1.4.3, tidyr v0.8.1, and tidyverse v1.2.1.

## Availability and requirements

Project name: SEPATHProject home page: https://github.com/UEA-Cancer-Genetics-Lab/sepath_tool_UEAOperating system(s): Linux-based high performance computing cluster environmentsProgramming language: Python 3, BashOther requirements: Python v3.5, Snakemake v3.13.3, Trimmomatic v0.36, Java v.8.0_51, bbmap v37.28, mOTUs2 v2.0.1, Kraken 1, Spades v3.11.1, Pysam v0.15.1License: GPL version 3 or later

## Supplementary information

**Additional file 1** Tab separated file containing the composition of all 100 bacterial simulated metagenomes.

**Additional file 2** Retention of bacterial reads using different depletion software.

**Additional file 3** Violin plots demonstrating performance in terms of F1-score, PPV and SSV for taxonomic ranks between Phylum and Species level on *n*=100 simulated datasets. (A) demonstrates performance of kraken when ran on raw reads with no read threshold. (B) Performance following the application of a read threshold (500 minimum) for each classification.

**Additional file 4** Coverage of contigs following metagenomic assembly on 99 simulated metagenomes. Higher values not shown in density plot.

**Additional file 5** Violin plot shows genus level performance with increasing minimum contig coverage filter but not to a large degree (Fig_S4.png). Tool used was Kraken on MetaSPAdes contigs.

**Additional file 6** An in depth look into Krakenuniq filtering parameters vs bacterial classification status for one simulated bacterial dataset.

**Additional file 7** A more in-depth look into contig parameters vs classification status for one of the viral datasets assembled using MetaSPAdes and classified using Kraken.

**Additional file 8** Scatter plot summarizing the constituents of all 100 simulated bacterial metagenomes. The y-axis demonstrates the number of bacterial reads in the datasets, whereas the number of human reads is shown on the x-axis. The number of species in each dataset is indicated by the color, darker points having less species. The distribution of each axis is shown in red.

**Additional file 9** Common metagenomics profile format (COMP) for viral simulations.

**Additional file 10** Review history.

## Abbreviations

BAM: Binary alignment map file format
HPC: High performance computing cluster
IQR: Interquartile range
NCBI: National Center for Biotechnology Information
PPV: Positive predictive value (precision)
RAM: Random access memory
SSV: Sensitivity (recall)
