# Supplementary figures and images for: SEPATH: benchmarking the search for pathogens in human tissue whole genome sequence data leads to template pipelines
Source: Genome Biol. 2019 Oct 22;20:208. doi: 10.1186/s13059-019-1819-8 (PMC6805339; doi:10.1186/s13059-019-1819-8)

# Human Depletion – Bacterial Read Removal

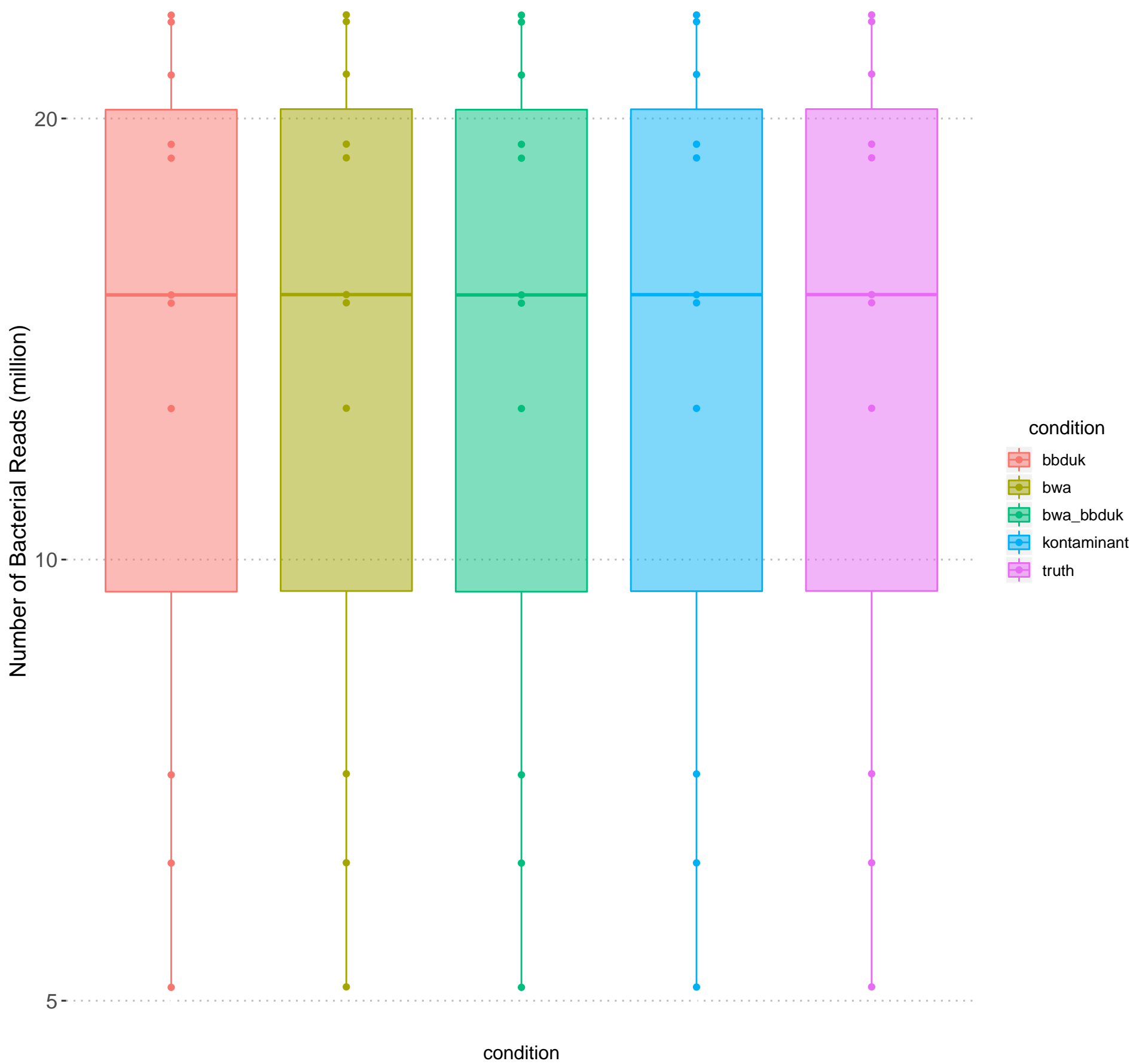

Supplement: Supplementary file 2 — Additional file 2 Retention of bacterial reads using different depletion software. [file 13059_2019_1819_MOESM2_ESM.pdf]

**A**

## Unfiltered Kraken

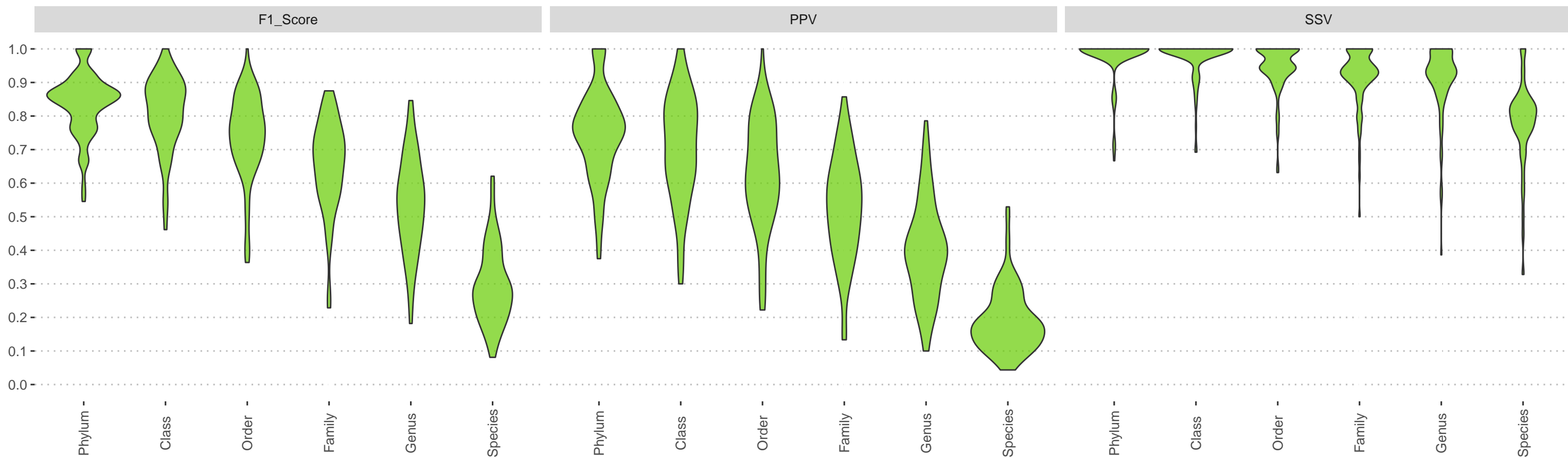**B**

## Read Filtered Kraken (500 read minimum)

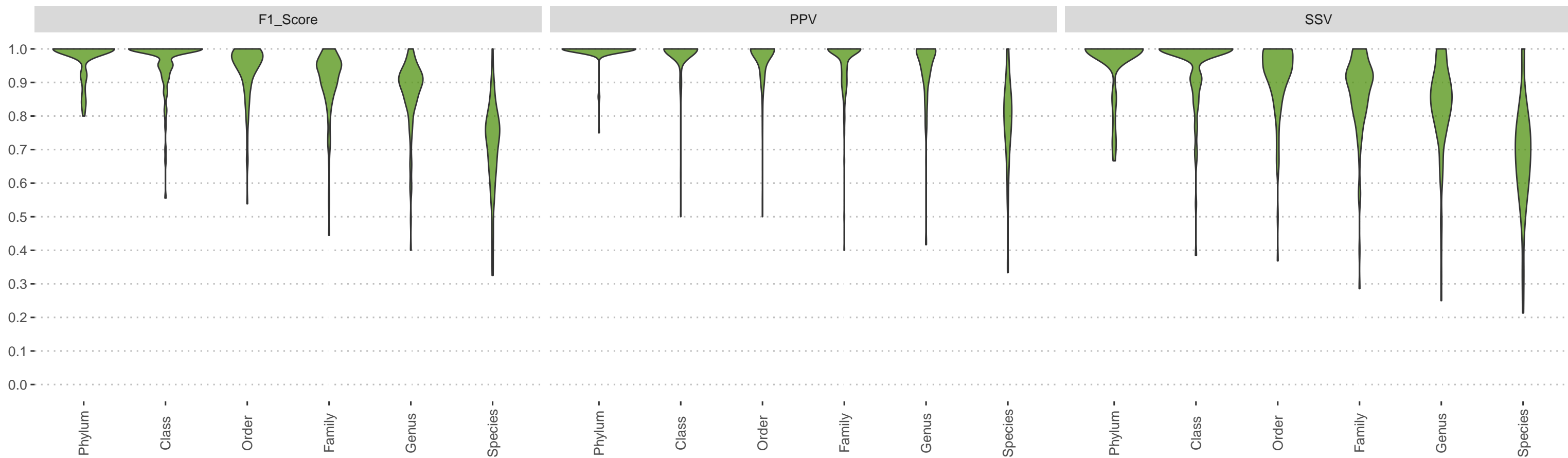

Supplement: Supplementary file 3 — Additional file 3 Violin plots demonstrating performance in terms of F1-score, PPV and SSV for taxonomic ranks between Phylum and Species level on n=100 simulated datasets. (A) demonstrates performance of kraken when ran on raw reads with no read threshold. (B) Performance following the application of a read threshold (500 minimum) for each classification. [file 13059_2019_1819_MOESM3_ESM.pdf]

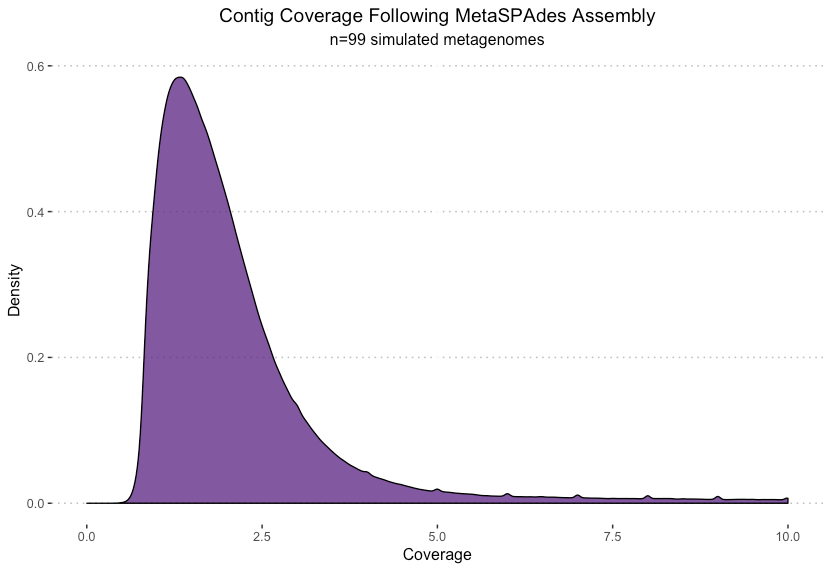

Supplement: Supplementary file 4 — Additional file 4 Coverage of contigs following metagenomic assembly on 99 simulated metagenomes. Higher values not shown in density plot. [file 13059_2019_1819_MOESM4_ESM.png]

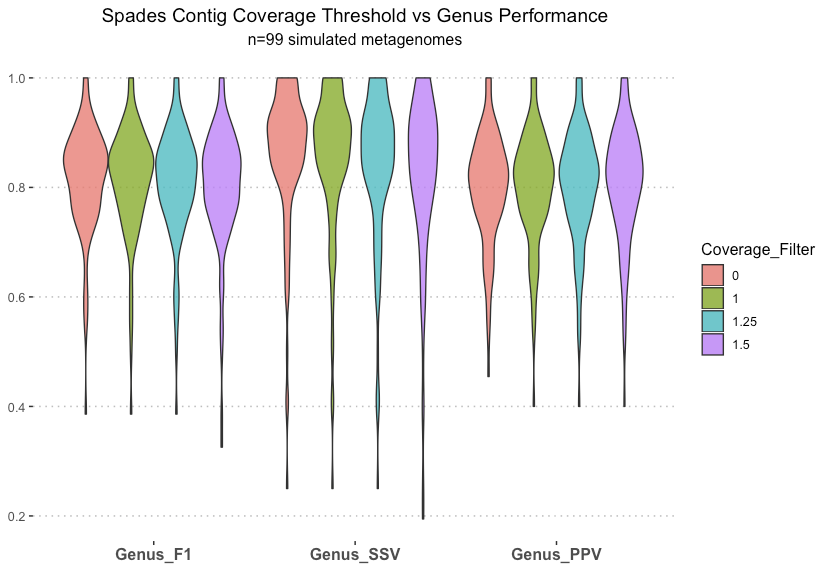

Supplement: Supplementary file 5 — Additional file 5 Violin plot shows genus level performance with increasing minimum contig coverage filter but not to a large degree (Fig_S4.png). Tool used was Kraken on MetaSPAdes contigs. [file 13059_2019_1819_MOESM5_ESM.png]

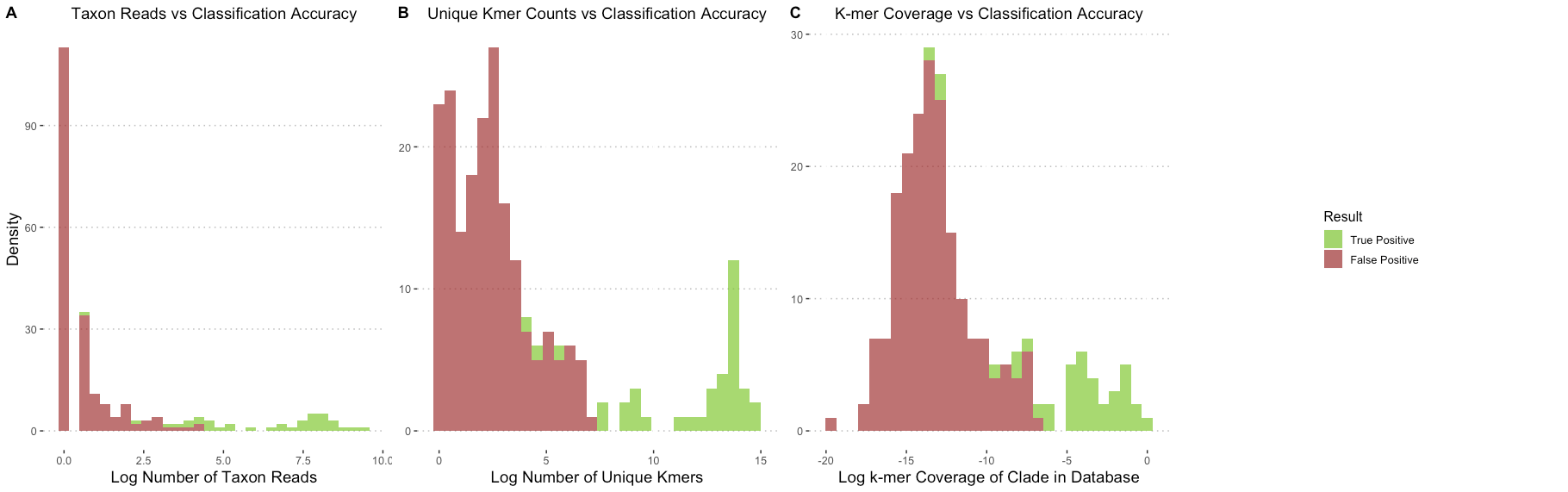

Supplement: Supplementary file 6 — Additional file 6 An in depth look into Krakenuniq filtering parameters vs bacterial classification status for one simulated bacterial dataset. [file 13059_2019_1819_MOESM6_ESM.png]

# Simulated Metagenomes Summary

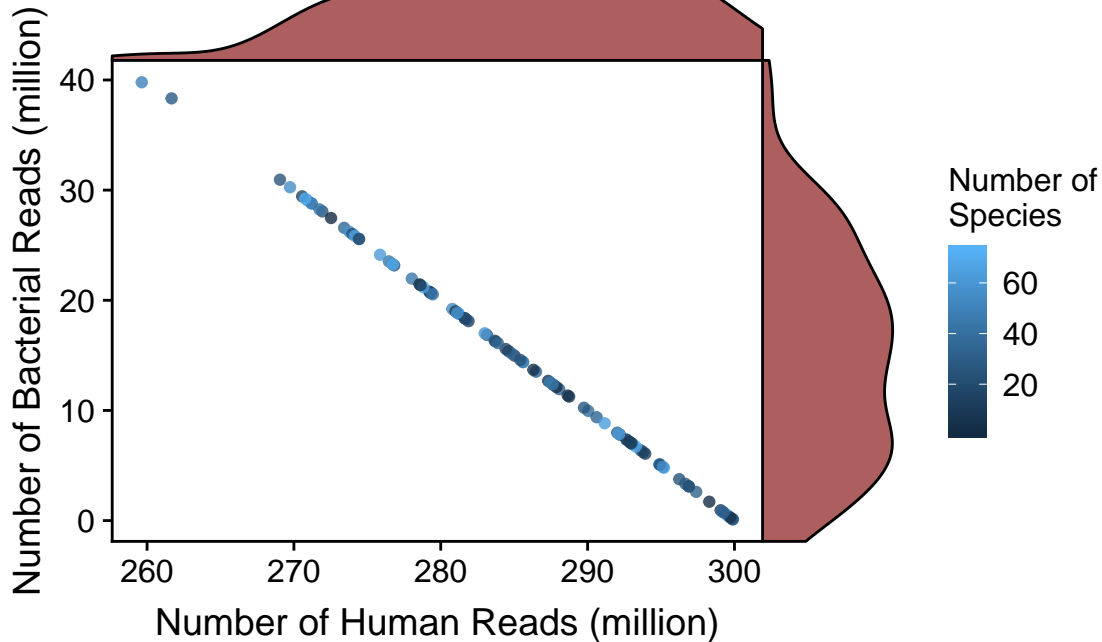

Supplement: Supplementary file 8 — Additional file 8 Scatter plot summarizing the constituents of all 100 simulated bacterial metagenomes. The y-axis demonstrates the number of bacterial reads in the datasets, whereas the number of human reads is shown on the x-axis. The number of species in each dataset is indicated by the color, darker points having less species. The distribution of each axis is shown in red. [file 13059_2019_1819_MOESM8_ESM.pdf]
