# Supplementary material for: SEPATH: benchmarking the search for pathogens in human tissue whole genome sequence data leads to template pipelines
Source: Genome Biol. 2019 Oct 22;20:208. doi: 10.1186/s13059-019-1819-8 (PMC6805339; doi:10.1186/s13059-019-1819-8)
Supplement: Supplementary file 7 — Additional file 7 A more in-depth look into contig parameters vs classification status for one of the viral datasets assembled using MetaSPAdes and classified using Kraken. [file 13059_2019_1819_MOESM7_ESM.pdf]

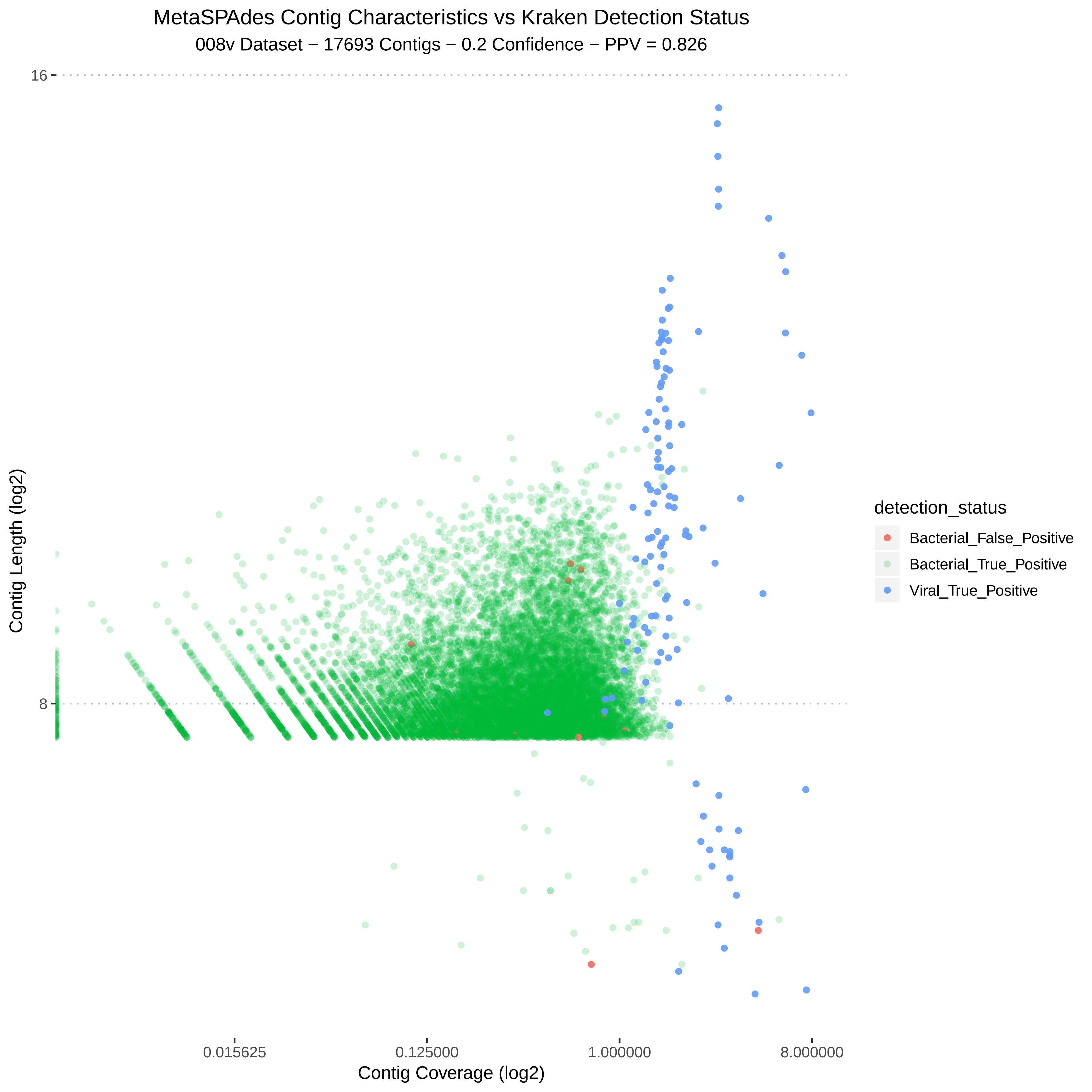
MetaSPAdes Contig Characteristics vs Kraken Detection Status
008v Dataset − 17693 Contigs − 0.2 Confidence − PPV = 0.826
16
8
Contig Length (log2)
0.015625
0.125000
1.000000
8.000000
Contig Coverage (log2)
detection_status
Bacterial_False_Positive
Bacterial_True_Positive
Viral_True_Positive
