# Supplementary material for: SEPATH: benchmarking the search for pathogens in human tissue whole genome sequence data leads to template pipelines
Source: Genome Biol. 2019 Oct 22;20:208. doi: 10.1186/s13059-019-1819-8 (PMC6805339; doi:10.1186/s13059-019-1819-8)
Supplement: Supplementary file 10 — Additional file 10 Review history. [file 13059_2019_1819_MOESM10_ESM.docx]

Review History

**First round of review**

**Reviewer 1**

**Comments to author:**

The theme of this manuscript is to develop a pipeline of available tools for identifying microbes from shotgun sequencing data of human tissues. The work has a potential to be valuable for a variety of clinical applications. However, there are several major issues with this work:

First, there is an apparently lack of novelty -- all bioinformatics tools are currently available, there is no new algorithms or softwares.

Second, it is fine to test the performance of this pipeline using artificial data by mixing various microbial reads with human ones, however, the readers would really appreciate if the authors could interrogate some real sequencing data from clinical samples.

Third, the authors put a lot of emphasis on cancer, which they refer as the clinical relevance of this work. This is perhaps an "over-sell" and inappropriate: i) the relationship between cancer and microbes is complex; whereas microbial involvement is true for some cancers such as gastric cancer, it is clearly not the cases for many, if not most cancers, notable examples include breast cancer, brain cancer; ii) as mentioned above, there are no real cancer tissue-derived shotgun data here. On the other hand, fast and accurate identification of microbes from human reads is crucial for many other clinical applications, such as identification of unknown and unculturable pathogens (including not just bacteria, virus, but also fungi, protozoa etc). Hence, the manuscript is perhaps better reorganized with the goal of efficient identifying microbes/pathogens from human tissues.

A minor issue:
It will also be interesting and possibly clinically valuable if the authors could test the performance using shorter reads data (i.e., 2X50bp; 2X100 bp).

**Reviewer 2**

**Comments to author:**

With the increasing whole genome sequencing data available from cancer tissues, identifying the pathogen sequences from these whole genome sequencing data is helpful to understand the cancers. The authors present here a comparison of over 70 combinations of tools and parameters to classifying the sequenced reads according to their source organism. Results showed that mOTUs2 and Kraken were the two tools with highest performance at genus level. A new tool called SEPATH was then constructed based on the comparison to detect pathogens in cancer sequence data. Though a large number of metagenome sequence classification tools exist, there is no calibration about their application to cancer metagenome analysis. This is important application direction for metagenome sequence classification methods. The pipeline can be a valuable tool for the research community. However, there are some serious problems with the analysis and results in this manuscript. Some of them are listed below. 

Major revisions: 

1.     Around line 7 of page 3, the authors mentioned that “there is a limited selection of computational pipelines available that allow users to get from raw reads to classifications/analysis”, this was not true. Almost all the metagenome sequencing classification tools can do sequence classification for reads, though the accuracy is associated to the length of the input sequences in addition to the percentage of reads that can be aligned to the reference database. 
2.      “it is unlikely to provide an accurate comparison for more niche areas of taxonomic classification such as ancient microbiome research or for intra-tumor metagenomics classification dominated by host sequences”, the accuracy of sequence classification methods depends heavily on the completeness of the reference database, which can be very different for different environmental samples. For example, the human gut metagenome sequence classification can be done very well since the reference genomes for gut bacteria are sufficient to do the sequence classification. However, this is not true for most metagenomes from other body sites. For intra-tumor metagenomics classification dominated by host sequences, the performance of sequence classification will depend on the body site as well as the relative abundance of bacteria to human. 
3.     For the step of host genome depletion, what is the impact of the completeness of the reference human genome? For example, the African human pan-genome project provided about 300 Mb novel sequences not included in the human reference genome. This may responsible for a big portion of the “non-host” sequences. 
4.     At page 8, the total number of simulated reads were set to 600 million, while in supplementary file 7, the total read number was fixed to 300 million. In addition, the numbers of bacterial reads were generated from a distribution with a mean of 28.4 million, while in Supplementary file 7, the mean of the bacterial read numbers was much smaller (maybe smaller than 20 million). 
5.     For the quality control step, the parameter for Trimmomatic program was not reasonable. For example, minimum read length set to 35 was kind of weird for a dataset with read length of 150. The quality of 15 over a sliding window of 4 was an old parameter setting for early sequencing platforms, such as those with read lengths about 50bp, and error rates at about a few percent.  
6.     For the human sequence depletion step, the results in Figure 1 was confusing. Figure 1A showed “number of human reads”, while in the figure legend it said the “remaining number of human reads”. It is unlikely that BWA-MEM can only align about half (more than 250 million human reads) of the simulated data to the human genome.
7.     For the comparison of sequencing classification methods, only mOTU2 and Kraken were evaluated. There are many other existing methods. Authors should explain why only these two were selected for evaluation. In addition, evaluation of sequence classification should be done at different taxonomy levels instead of just at genus level. 
8.     The limitation of SEPATH should be discussed. For example, it cannot do sequence classification at strain level, which is becoming more and more important in recent years. 
9.     In Figure 3, the R2 values were based on relative abundance and read number for mOUTs2 and Kraken respectively. These two values are not comparable. Authors should use the same criterion (either abundance or read number) for two methods. 
10.     In Figure 3, what are these 2,084 and 2,021 “True Positive Results”? There was no explanation. 
11.     For virus sequence classification, only contigs based sequence classification results were reported in Table 1 though running Kraken with reads was mentioned in the main text. In addition, it’s better to include the recall and specificity of species identification in Table 1. 
12.     The installation of SEPATH pipeline is not straightforward. It can be improved. 
13.     All tests were based on simulated sequencing data. It is better to include some real sequencing data sets to show the performance of different methods. 
14.     Post-classification filtering was mentioned multiple times in the manuscript. However, there was no description about the exact meaning of Post-classification filtering. 

Minor revisions: 
1.     In Figure 6 and in the main text, the input file for SEPATH was in FASTAQ format, while in Methods part the data generated by BEAR was in FASTA format. Which format did SEPATH support? 
2.     Definition equations for recall (1) and precision (2) are the same. The equation for precision should be TP/(TP+FP).

**Authors Response**

**Point-by-point responses to the reviewers’ comments:**

Reviewer #1

1.1. “the readers would really appreciate if the authors could interrogate some real sequencing data from clinical samples.”

*Response: We agree that the manuscript would benefit from the inclusion of real data. The revised manuscript submission includes analysis of real sequencing data from clinical samples. We have created a new figure to summarise our findings (figure 7). In this analysis we trial both Kraken and mOTUs2 on quality-trimmed human-depleted sequencing reads. Both tools detected Helicobacteri in stomach cancer as expected (Plummer et al. 2016) as well as a range of other interesting genera such as Lymphocryptovirus (McLaughlin-Drubin and Munger 2008) and more. Kraken analysis of cervical cancer samples revealed that Alphapapillomavirus (genera of HPV) is detected with a higher average number of reads compared with other taxa. A range of interesting other taxa appeared to be present in classifications using both tools including: Escherichia, Prevotella, Porphyromonas Propionibacterium and Fusobacterium. Interestingly Treponema is highlighted as present in two cervical cancer samples with a higher average number of reads which could represent an interesting incidental finding. Low numbers of taxa were detected in Medulloblastoma when ran with mOTUs2, and the taxa that were detected were all of a much lower read count compared with the other cancers. This is a good finding for SEPATH as the brain is currently presumed to be mostly sterile in healthy individuals; although currently a subject of debate (Salzberg et al. 2016).*

*Changes:*

*- A new results section has been added to highlight some of the key findings and how they pertain to the practical applicability of the pipelines presented in the manuscript. (page 7 line 19)*

*- These new results are have resulted in extra discussion (page 9 line 23).*

*- These results have also been added to the abstract (page 1 line 34)*

*- Methods have been added pertaining to this extra analysis (page 11 line 6)*

1.2. “all bioinformatics tools are currently available, there is no new algorithms or softwares.”

*Response: While it is correct that the novelty in this manuscript is not due to new algorithms, we do address a deficiency within the scientific community regarding the optimal combination of computational metagenomic approaches in host dominated whole genome sequence data. This is a practical approach that we hope will be of considerably utility to the scientific community.*

*Changes:*

*- None*

1.3. “the authors put a lot of emphasis on cancer, which they refer as the clinical relevance of this work. … On the other hand, fast and accurate identification of microbes from human reads is crucial for many other clinical applications, such as identification of unknown and unculturable pathogens (including not just bacteria, virus, but also fungi, protozoa etc). Hence, the manuscript is perhaps better reorganized with the goal of efficient identifying microbes/pathogens from human tissues.”

*Response: This is a very useful comment. The reviewer is correct that we had made the emphasis on cancer. There is currently considerable interest in the potential interaction between bacteria and cancer development and we have been involved in projects including the International Cancer Genome Consortium and Genomics England where there is ongoing interest. For prostate cancer, for example, there is still a big debate on whether or not pathogens are involved (De Marzo et al. 2007; Feng et al. 2019; Hochreiter et al. 2000). However the reviewer raises the important issue that the identification of pathogens within host-dominated sequencing data is important in other contexts.*

*This could include:*

*- Identification of pathogens involved in the aetiology of non-cancer disease.*

*- The identification of known harmful (life threatening) pathogens.*

*Accordingly, we have broadened out the application of the work.*

*Changes:*

*- The abstract has been adapted to reduce focus on cancer*

*- ‘Cancer’ has been removed from the title of the manuscript*

*- Various minor text changes throughout the manuscript to encompass the use of this pipeline on human tissue over just cancer tissue.*

1.4 “It will also be interesting and possibly clinically valuable if the authors could test the performance using shorter reads data (i.e., 2X50bp; 2X100 bp).”

*Response: While we agree that it might be an interesting exercise to look at different sequence read lengths this would, we believe, have limited use practically as the majority of major cancer sequencing projects now sequence at lengths of 2 x 150bp e.g. Genomics England, ICGC ARGO recommendations etc. That said the real datasets that we have investigated originated from 2x100bp reads. We have adapted our parameters as required and discussed this in the text.*

*Changes:*

*- In the methods we have mentioned how the parameters were adjusted for the shorter read technology (page 11, line 17)*

*“mOTUs2 was ran for genus level analysis on the same reads using two marker gene minimum and a non-standard minimum alignment length of 50 to account for shorter read length. Kraken files had a minimum read threshold applied of 100 reads for each classification and mOTUs2 results were left unfiltered.”*

*- This was also mentioned in the revised discussion (page 9 line 25) “ Kraken appears to be more sensitive than mOTUs in this real data, possibly due to the differing parameters used due to the shorter read lengths seen (2x100bp in real sample data compared to 2x150bp in simulated data). This suggests that performance may vary depending on input data quality and adapted parameters”*

Reviewer #2: Major revisions:

2.1. Around line 7 of page 3, the authors mentioned that “there is a limited selection of computational pipelines available that allow users to get from raw reads to classifications/analysis”, this was not true. Almost all the metagenome sequencing classification tools can do sequence classification for reads, though the accuracy is associated to the length of the input sequences in addition to the percentage of reads that can be aligned to the reference database.

*Response: We thank the reviewer for this comment and the opportunity to clarify this remark. When we refer to ‘raw’ reads, we mean reads directly from the sequencing machine including a majority of host originating reads. The steps presented in this manuscript quality control sequencing reads and removal of host contamination prior to submitting them to metagenomic classification. Most published tools for metagenomic classification assume high-quality input sequences. We have changed the document to reflect this.*

*Changes:*

*- We clarified the requirements of pipelines for this purpose (page 3 lines 9-10) “Although there is a large number of tools available purely for sequence classification, at the time of writing there is a limited selection of computational pipelines available that process data optimally and produce classifications from raw reads with all appropriate steps including quality control.*

2.2. “it is unlikely to provide an accurate comparison for more niche areas of taxonomic classification such as ancient microbiome research or for intra-tumor metagenomics classification dominated by host sequences”, the accuracy of sequence classification methods depends heavily on the completeness of the reference database, which can be very different for different environmental samples. For intra-tumor metagenomics classification dominated by host sequences, the performance of sequence classification will depend on the body site as well as the relative abundance of bacteria to human.

*Response: The choice of reference databases is one of the largest factors affecting metagenomic tool performance. Additionally, searching for pathogens in human tissue sequence data is confounded by the relative and varying dominance of host reads and a very small proportion of reads originating from microorganisms. Completeness of the reference databases are beyond our control but this paper addresses how to optimally detect known microorganisms in host dominated sequence data.*

*In response to referees comments we would note.*

*1. The simulations that we have already performed represents a spectrum of human: micro-organism ratios that we expect to find in tissue. The proportion of bacterial reads to human reads ranges from as little as 0.033% to 15.32% (supplementary file 7).*

*2. The bacteria we have selected for our simulations were identified across a range of bodily sites (supplementary file 1).*

*3. We have now applied the mOTUs2 and Kraken pipelines to stomach, cervical and medulloblastoma samples showing utility in a range of tissues.*

*4. Tools employed in this analysis, particularly for Kraken and mOTUs2 utilise up to date (as reasonably possible) information of the tree of life. mOTUs2 uses a curated database that is built from not only all available high-quality reference sequences, but also incorporates information from metagenome assembled genomes from environmental samples as well as both disease and normal states in human. This renders mOTUs2 as a state-of-the-art tool for this type of investigation.*

*5. Kraken provides unclassified reads/contigs which can be investigated, or these sequences will obtain classifications higher up the taxonomic tree. mOTUs2 reports a ‘-1’ classification, which denotes the relative abundance of (bacterial) taxa that have been identified but have not been associated with any taxonomic labels. It is possible to extract the sequencing reads that align against a particular taxonomic ranking for subsequent analysis.*

*Changes:*

*- We have included additional discussion to cover these points and limitations in the databases used are also mentioned in the discussion (Page 8 Line 25, page 9 line 3, page 9 line 18).*

*“mOTUs2 has been developed with quantitative abundance in mind. It intuitively estimates the proportion of sequences estimated to originate from unknown taxa (denoted by ‘ -1'} in mOTUs2 reports), and adjusts abundance values from detected clades accordingly to account for this”*

*“Although both tools are state-of-the-art at the time of writing, they are likely to contain biases in terms of what they are able to classify, which pertains to previous sequencing efforts of the sampling site. The human gut microbiome for example is currently believed to be better characterised than other body sites.”*

*“Benchmarking tools and databases as they emerge is an important task as they greatly influence performance. It is hoped that utilities presented here will assist future benchmarking efforts.”*

2.3. For the step of host genome depletion, what is the impact of the completeness of the reference human genome? For example, the African human pan-genome project provided about 300 Mb novel sequences not included in the human reference genome. This may responsible for a big portion of the “non-host” sequences.

*Response: We thank the reviewer for making this excellent point. In the revised manuscript we have performed human read depletion analysis following the inclusion of sequences from the African human pan-genome project data (Sherman et al. 2019).*

*We find that the inclusion of the additional human sequences did not significantly reduce the number of bacterial reads. In the interest of capturing more variation in the human genome, we recommend use of these sequences in the revised manuscript (figure 1) and use them for additional analysis.*

*Changes:*

*- Figure 1 has been adjusted to include the African pan-genome project data as an additional depletion parameter (figure legend has been adjusted accordingly).*

*- The added analysis of real data employed the newly generated database containing African pan-genome sequences.*

*- Various changes throughout the text (page 5 line 2, page 11 line 13)*

*“To investigate using a contaminant database capturing a higher degree of human sequence variation, we also investigated the inclusion of additional human sequences from a recent analysis into the African 'pan-genome'. Including these extra sequences removed slightly more bacterial reads, but was a very minor effect (Figure 1C).”*

*“SEPATH standard parameters but with a database consisting of human reference genome 38, African 'pan-genome' project sequences and COSMIC cancer genes as previously mentioned”*

2.4. At page 8, the total number of simulated reads were set to 600 million, while in supplementary file 7, the total read number was fixed to 300 million. In addition, the numbers of bacterial reads were generated from a distribution with a mean of 28.4 million, while in Supplementary file 7, the mean of the bacterial read numbers was much smaller (maybe smaller than 20 million).

*Response: Thank you for highlighting this point. We agree that the previous submission was not clear on this point.*

*In our simulations there were 300 million pairs of reads and 600 million reads. Supplementary file 7 shows the number of paired reads in all files.*

*The number of reads for each simulated dataset was selected randomly from a normal distribution with mean 28.4 million and sd 20.9 million. This sometimes will generate negative values (see plot below based on simulated data) and in this situation another random value was chosen. This skews the mean of the output values to be higher than the initial value of 28.4 million. The actual mean of bacterial reads was 14.93 million paired reads (29.87 million total single reads). Supp fig 7 shows the number of paired reads hence the lower amount.*

*We have altered the manuscript in the following way to clarify this:*

*- In the methods for metagenome simulation we have clarified that the values discussed represent total number of reads originating from both pairs (page 10, lines 12,13,6) “Firstly, the number of total bacterial reads (in both pairs) was generated by a random selection of positive values from a normal distribution function with a mean of 28,400,000 and a standard deviation of 20,876,020. The number of human reads in the sample was set to the difference between this number and 600 million (the total number of reads in both pairs).”*

2.5. For the quality control step, the parameter for Trimmomatic program was not reasonable. For example, minimum read length set to 35 was kind of weird for a dataset with read length of 150. The quality of 15 over a sliding window of 4 was an old parameter setting for early sequencing platforms, such as those with read lengths about 50bp, and error rates at about a few percent.

*Response: We are sorry to hear that the reviewer does not find the parameters reasonable. The minimum read length was deliberately set low to preserve as much high quality pathogenic reads as possible when there is such a small amount of data. This is still larger than the minimum kmer length required for either Kraken or BBDuk. In practice, this would make little difference with ~5% of reads being smaller than 50bp after the predominant trimming parameters and human depletion used (density plot below). With a BBDuK k-mer length of 30 and a minimum read length of 35, 3/6 k-mers would need to be exact matches to the human contaminant database for removal. We believe that this is sufficient evidence that the read should be retained. Furthermore mOTUS2 would not align successfully to shorter reads (default minimum alignment of 75), and confidence thresholding in kraken means that multiple k-mers need to assign to the same taxa for a successful classification. For read lengths of 50 (95% of reads) and a kraken k-mer length of 31 and a kraken confidence threshold of 0.2, this equates to a minimum of 4 k-mers being an exact match to a particular taxa for classification. We believe these parameters to be sufficient which is reflected in the high tool performance.*

*The pipelines in SEPATH have default trimming parameters that are set intentionally on the low side to account for lower quality datasets and to preserve pathogenic reads. These parameters are completely adjustable for the user depending on the requirements of their individual datasets.*

*Changes:*

*- We have clarified that users should adjust trimming parameters according to their own data. (page 10, line 37)*

*- We have clarified that we set the parameters low to prevent removal of potentially pathogenic reads (page 10 line 37-38) “SEPATH has trimming parameters set as default that prevent any excessive removal of data (including any reads that may be pathogenic), but these should be adjusted according to the nature of the data being analysed.”*

*- We have added text to the discussion regarding input data quality (page 9 lines 27-29) “This suggests that performance may vary depending on input data quality and adapted parameters”*

2.6. “For the human sequence depletion step, the results in Figure 1 was confusing. Figure 1A showed “number of human reads”, while in the figure legend it said the “remaining number of human reads”. It is unlikely that BWA-MEM can only align about half (more than 250 million human reads) of the simulated data to the human genome.”

*Response: We apologise for the confusion caused in Figure 1 and have adjusted the axis titles in the revised manuscript to improve clarity. BWA-MEM preserved all pairs of reads where at least one read was listed as unmapped. This was done deliberately to preserve as many as possible of the pathogenic reads. Given that BWA-MEM aligned BAM files are usually how data is presented from large scale high throughput studies we find this is a useful base-line to compare further human read depletion steps.*

*Changes:*

*- Figure 1A has been adjusted so that the y-axis reads ‘remaining number of human reads’ - The conservative approach taken has been clarified in the text (page 4 line 25).*

*“BWA-MEM is used as a baseline and parameters were set to be as preservative as possible of any potential non-human reads”*

2.7a. “For the comparison of sequencing classification methods, only mOTU2 and Kraken were evaluated. There are many other existing methods. Authors should explain why only these two were selected for evaluation.

*Response: We evaluated 6 classification tools in the primary comparison that are representative from the range of different approaches in this area (see Figure 2). These tools are: MetaPhlAn2, Kaiju, Gottcha and Centrifuge in addition to Kraken and mOTUs2. We also provide a similar benchmark on Pathseq on a reduced selection of datasets. Diamond and BLAST were also used as part of this analysis in analysing contigs. Kraken and mOTUs2 were taken forward for further investigation because they were the highest performing tools in the tests presented here.*

*Changes: - None*

2.7b “In addition, evaluation of sequence classification should be done at different taxonomy levels instead of just at genus level.”

*Response:The choice of taxonomic level is an interesting one. In the manuscript we relay the findings of recent analysis that demonstrates the instability of species level classification with the rapidly growing size of sequence repositories (Nasko et al. 2019). From prior analysis we have shown that Genus level to be consistently highly performing. Whilst we are unable to demonstrate all performance values over every taxonomic level for every test condition, we do provide more of a rationale as to why we selected Genus level for investigation in the revised version of the manuscript.*

*Changes:*

*- Supplementary file 8 added that demonstrates performance across taxonomic levels for a sample tool. - Added text to reference the figure demonstrating performance across taxonomic levels (page 9 line 9)*

*“For bacterial classification, we noted a higher performance at taxonomic levels above genus level but performance appears to drop at species level (supplementary file 8).”*

2.8. The limitation of SEPATH should be discussed. For example, it cannot do sequence classification at strain level, which is becoming more and more important in recent years.

*Response: We thank the reviewer for their comment. We have added details in the manuscript that better describes the limitations.*

*Concerning the strain level classification, while this is possible with the pipeline the data sets in this domain make this problematic. The requirements of data and tools to make accurate classifications at strain level are quite restrictive. The input data needs to be of a sufficient amount (which limits low abundance applications) and quality to be able to distinguish strains that are often very similar. In host dominated sequence data the quantity and quality is not there. The optimal taxonomic level is at genus level as explained in response 2.7b.*

*Given better input data SEPATH tools can be used for strain level classification but this is beyond the scope of this work. SEPATH using Kraken contains adjustable databases (our database actually allows for some strain level classification should the input data permit it).*

*mOTUs2 allows to output the alignment files. From these BAM files, and using scripts provided by mOTUs2, it is possible to identify strains.*

*The discussion in the reviewed manuscript has been adjusted to include this perspective.*

*Changes:*

*- Additional discussion has been added to reflect these points (Page 9 line 9) “For bacterial classification, we noted a higher performance at taxonomic levels above genus level but performance appears to drop at species level (supplementary file 8). We urge caution when working at the species level on this type of data due to this combined with the instability of species level classification. At lower taxonomic levels, the retention of BAM files from mOTUS2 could theoretically allow for subsequent investigations at more specific taxonomic nodes (such as strain level) by investigating single nucleotide variation. Kraken also automatically produces sub-genus level classifications where the input data and reference database permits. Validating performance at these taxonomic levels would require extensive performance benchmarking which has not been conducted here. Benchmarking tools and databases as they emerge is an important task as they greatly influence performance. It is hoped that utilities presented here will assist future benchmarking efforts.”*

2.9. In Figure 3, the R2 values were based on relative abundance and read number for mOUTs2 and Kraken respectively. These two values are not comparable. Authors should use the same criterion (either abundance or read number) for two methods.

*Response: Please find included in the revised manuscript an updated version of figure 3. We have reanalysed all 100 samples using mOTUs2 with read count outputs, verified that the performance obtained using read counts vs relative abundance is identical (violin/dot plot below) and subsequently reproduced the figure. Extra analysis scripts have been added to the analysis github repository.*

*Changes: - The header for the relevant results section has been changed to ‘Bacterial Proportion Estimation’ (page 5, line 31) - In the text. Mentions of ‘relative abundance’ regarding motus have been adapted to ‘quantitative measures’ to fit the altered metric (page 5 lines 36-38, page 8 lines 25,41 page 9, line 44)*

2.10. In Figure 3, what are these 2,084 and 2,021 “True Positive Results”? There was no explanation.

*Response: An explanation of “True Positive Results” was provided in the legend for figure 3. It is only possible to compare quantitative metrics for true positive classifications. On our simulated datasets, we obtained 2,084 true positive classifications for mOTUs2 and 2,021 true positive results for Kraken. Changes:*

*- We have removed these numbers from the graph subtitles and only mentioned sample sizes in the legend to avoid any confusion in the revised figure.*

2.11. “For virus sequence classification, only contigs based sequence classification results were reported in Table 1 though running Kraken with reads was mentioned in the main text. In addition, it’s better to include the recall and specificity of species identification in Table 1.” Response: Thank you for your comment. To improve the manuscript we have created a new figure (figure 5) in place of table 1 which provides a much more in-depth analysis of bacterial and viral performance on: - Reads and metagenome assembled contigs - With and without the application of post-classification filtering parameters. As requested, these results have been presented in terms of sensitivity and PPV. Changes: - Figure 5 created and the corresponding legend has been included. - Table 1 removed - Adjustments to the text have been made to reflect the new results (page 6, lines 35-46) “Kraken was run on either quality trimmed reads or contigs following metaSPAdes assembly (see Methods). Kraken correctly identified 8/10 virus species from reads as input with post-classification filtering. When using contigs and no filtering strategies, 7/10 species were detected with no viral false positive results (Figure 5B). Filtering by minimum number of contigs removed the majority of viral classifications. The effect of filtering on viral species classification was not reflected in classification of bacterial genera (Figure 5A).” 12. The installation of SEPATH pipeline is not straightforward. It can be improved.

*Response: We have made the following adaptations in response to your comment to improve the installation: 1- sample_sepath.py has been created and can be found in the tutorial directory, instructions for usage are provided on the github repository. Please note that this downloads small databases so it can be tested on a standard laptop computer. Other required files to repeat our analysis, such as files from the COSMIC database are openly available for the moment, but we do not currently have permission to share this data. 2- We have created a containerised singularity environment for which our pipelines can be executed within. A sample version of SEPATH can be ran with the following lines (with git and singularity):*

*git clone git@github.com:UEA-Cancer-Genetics-Lab/sepath_tool_UEA.git cd sepath_tool_UEA/tutorial/ singularity pull library://agihawi/default/sepath singularity shell sepath_latest.sif ./sample_sepath.py*

*3- Dependencies can also be installed by creating a conda environment. We provide details in the tutorial on the github on how to install these. Using Conda or singularity also eliminates the need for administrator access on a cluster environment.*

*To fully utilise available resources, cluster arguments must be adjusted, which depends heavily on the resources available to the researcher and is highly variable. So please be aware that if you are using sample_sepath.py, it will not run as efficiently as the main SEPATH pipeline and will require >55GB memory to use the human reference database as a contaminant. If you would like assistance with implementing SEPATH on your local high-performance computing cluster, please get in touch or submit an issue on the SEPATH github page. We would be happy to help.*

*Changes: - Text has been added describing the use of conda and singularity to install software dependencies (page 8 line 6). “All software dependencies for SEPATH can easily be installed via conda” - Additional changes to the github repository referenced in the manuscript (as discussed)*

13. All tests were based on simulated sequencing data. It is better to include some real sequencing data sets to show the performance of different methods.

*Response: Thank you for your comment. As mentioned in response 1.2, we have included analysis of real data in the revised manuscript. These results support the effectiveness of this pipeline.*

14. Post-classification filtering was mentioned multiple times in the manuscript. However, there was no description about the exact meaning of Post-classification filtering.

*Response: Post-classification filtering involves any filtering parameters that happen on the results produced by a metagenomic classification tool. We have made this clearer in the revised version of the manuscript. Changes: - Added clarity regarding post-classification filtering (page 6, line 6) “Post-classification filtering involves applying criteria to remove low quality classifications from taxonomic results.”*

Minor revisions:

1. In Figure 6 and in the main text, the input file for SEPATH was in FASTAQ format, while in Methods part the data generated by BEAR was in FASTA format. Which format did SEPATH support?

*Response: SEPATH has been designed to function from either BAM or FASTQ input files. The text in the methods refers to the generation of simulated data. FASTA files were generated prior to the application of an error model, which was used to convert the FASTA simulations to FASTQ. We have adjusted the text in the revised manuscript to further clarify this.*

*Changes:*

*- Very minor text adaptation to solidify the input file formats used. (page 7 line 45)*

*- “All blocks can be run together or separately and uses either BAM of FASTQ input files.”*

*- Figure 8 has been simplified and ‘input’ has been removed from FASTQ files*

2. Definition equations for recall (1) and precision (2) are the same. The equation for precision should be TP/(TP+FP).

*Response: Thank you for noticing this. We have edited the equation in the revised manuscript. Further Changes:*

*- Use of these tools on real data has revealed important considerations between the two tools. Text has been added to the discussion regarding this matter.*

*- Additional tools and R packages have been utilised for the production of the revised manuscript (page 11, lines: 12,19-20)*

*- Added availability of data and materials (page 12, line 10)*

*- Added author contributions for extra analysis (page 12 line 18)*

*- Minor text changes as highlighted to improve readability*

*- Additional correspondence email address added (page 1 line 14)*

*- Simplified author address (page 1 line 14, page 13 line 16)*

*References:*

*De Marzo AM, Platz EA, Sutcliffe S, Xu J, Gronberg H, Drake CG, et al. Inflammation in prostate carcinogenesis. Nat Rev Cancer. 2007;7(4):256-69.*

*Feng Y, Ramnarine VR, Bell R, Volik S, Davicioni E, Hayes VM, et al. Metagenomic and metatranscriptomic analysis of human prostate microbiota from patients with prostate cancer. BMC Genomics. 2019;20(1):146.*

*Hochreiter, W. W. and Duncan, J. L. and Schaeffer, A. J. Evaluation of the bacterial flora of the prostate using 16s rRNA gene based polymerase chain reaction. J. Urol. 2000;163(1):127-130*

*Nasko, D.J., Koren, S., Phillippy, A.M., Treangen, T.J. Refseq database growth influences the accuracy of k-mer-based lowest common ancestor species identification. Genome Biol 19(1) (2018).*

*McLaughlin-Drubin M, Munger K. Viruses Associated with Human Cancer. Biochim Biophys Acta. 2008;1782(3):127-50.*

*Plummer M, de Martel C, Vignat J, Ferlay J, Bray F, Franceschi S. Global burden of cancers attributable to infections in 2012: a synthetic analysis. The Lancet Global Health. 2016;4(9):e609-e16.*

*Salzberg SL, Breitwieser FP, Kumar A, Hao H, Burger P, Rodriguez FJ, et al. Next-generation sequencing in neuropathologic diagnosis of infections of the nervous system. Neurol Neuroimmunol Neuroinflamm. 2016;3(4):e251.*

*Sherman, R.M. Forman, J. Antonescu, V. Puiu, D. […] and S.L. Salzberg. Assembly of a pan-genome from deep sequencing of 910 humans of African descent. Nature Genetics 51, 30-35 (2019).*

**Second round of review**

**Reviewer 1**

I thank the authors for devoting tremendous effort in addressing the comments and am pleased to find that the manuscript has been greatly improved. One issue I have is with the test results of real sequecing data of clinical samples (Figure 7): as we can clearly see, Kraken and mOTUs2, which according to the authors are the two best performing methods, produced very different results. Although this is somewhat to be expected giving their underlying bioinformatic approaches, the issue should not be simply ignored or avoided. As a matter of fact, this illustrates that the very problem that the authors embark on solving with this pipeline--“rendering some clades underrepresented or entirely absent --Ln 42 ”-- remains in place. As such, I would recommend that this limitation of the pipeline is adequately discussed and if possible, some tangible solutions are offerred, e.g., under what circumstances is Kraken or mOTUS2 preferred etc.

The manuscript has been greatly improved and should be ready for publication if the issue of conflicting results of clinical samples derived from Kraken and mOTUs2 (Fig 7) is adequately addressed.

**Reviewer 2**

The manuscript has been revised and improved significantly. A few issues are listed below. 

1.     The description about the current progress in metagenome sequence classification was still misleading. Most classification method can do the work, but their performance on host sequences dominated datasets has not been evaluated.

2.     It’s better to change “host contamination” to some other words since the human genome sequences were the target sequences for the sequencing projects, in which the human sequences are not contamination. 

3.     All the bacteria and virus involved in the simulated datasets have their genome sequences available. However, in most real metagenome datasets, majority of the bacterial or virus reads were from unknown organisms with no genome sequence available. This should be addressed in the paper.

**Authors Response**

**Point-by-point responses to the reviewers’ comments:**

Reviewer Comments:

**Reviewer #1:**

1.1 “One issue I have is with the test results of real sequecing data of clinical samples (Figure 7): as we can clearly see, Kraken and mOTUs2, which according to the authors are the two best performing methods, produced very different results. Although this is somewhat to be expected giving their underlying bioinformatic approaches, the issue should not be simply ignored or avoided. As a matter of fact, this illustrates that the very problem that the authors embark on solving with this pipeline--“rendering some clades underrepresented or entirely absent --Ln 42 ”-- remains in place. As such, I would recommend that this limitation of the pipeline is adequately discussed and if possible, some tangible solutions are offerred, e.g., under what circumstances is Kraken or mOTUS2 preferred etc.”

*It is correct that the use of tools on real data presents some interesting differences. To meet this concern and the final concern of reviewer 2 (below) we have expanded the final paragraph of the discussion. We expand on how mOTUs2 has proven useful in faecal metagenomics and suggest that an apparent higher sensitivity on these samples compared to tissue data is likely due to differences in microbial genome coverage and the use of sequencing methodology that is optimised for bacterial detection. We therefore recommend Kraken as the predominant investigatory tool but suggest that mOTUs2 has a useful role in confirmation of results and is better suited to investigate ‘unknown’ taxa.*

**Reviewer #2:**

2.1. The description about the current progress in metagenome sequence classification was still misleading. Most classification method can do the work, but their performance on host sequences dominated datasets has not been evaluated.

*We apologise that the reviewer found our adjustment misleading. Due to differences in genome size, an equal mixture of human and bacterial cells yields 99.9% human DNA (Salzberg et al. 2016). Therefore, running various analysis steps on this many reads would simply not be practical. To address this concern and to help clarify the point, we have added in the mention of ‘throughput’ with regards to the optimal processing of data.*

*“at the time of writing there is a limited selection of computational pipelines available that process data optimally with high-throughput and produce classifications from raw reads with all appropriate steps including quality control.”*

2.2. It’s better to change “host contamination” to some other words since the human genome sequences were the target sequences for the sequencing projects, in which the human sequences are not contamination.

*Thank you for your comment. The reviewer is correct in that using the term ‘host contamination’ is somewhat of a misnomer. In the resubmission, the presence of ‘host contamination’ has been diminished. We refer to ‘BBDuK’ instead of ‘contaminant’ databases and ‘human’ instead of ‘contaminant’ depletion.*

2.3. All the bacteria and virus involved in the simulated datasets have their genome sequences available. However, in most real metagenome datasets, majority of the bacterial or virus reads were from unknown organisms with no genome sequence available. This should be addressed in the paper.

*Thank you for your comment. To address this concern we have adjusted the final paragraph in the discussion which we hope addresses this comment along with the comments of reviewer 1. In this paragraph we expand upon the use of mOTUs2 in capturing a higher degree of ‘unknown’ organisms or microbial dark matter compared with Kraken and we also state how standard Kraken databases are unlikely to provide insight into any of these unknown reads.*

*References: Salzberg SL, Breitwieser FP, Kumar A, Hao H, Burger P, Rodriguez FJ, et al. Next-generation sequencing in neuropathologic diagnosis of infections of the nervous system. Neurol Neuroimmunol Neuroinflamm. 2016;3(4):e251.*
